# Choosing the right treatment for patients with a severe course of chronic non-bacterial osteomyelitis (CNO) - pamidronate or TNF-α blockade?

**DOI:** 10.1186/1546-0096-13-S1-P190

**Published:** 2015-09-28

**Authors:** H Morbach, A Schnabel, N Bruck, A Holl-Wieden, H Girschick, C Hedrich

**Affiliations:** 1 University Childrens Hospital, Pediatric Rheumatology, Würzburg, Germany; 2 University Childrens Hospital, Pediatric Rheumatology, Dresden, Germany; 3 Vivantes Hospital im Friedrichshain, Childrens Hospital, Berlin, Germany

## 

Chronic non-bacterial osteomyelitis (CNO) is an inflammatory, non-infectious disorder of the skeletal system with unknown aetiology. Therapeutic options are NSAIDs, steroids and DMARDs (MTX or sulfasalazine). However, a considerable number of patients have a severe disease course and bisphosphonates or TNF-α blockade might be a therapeutic option.

We performed a multicentre, retrospective chart review of all patients diagnosed with CNO in two paediatric rheumatology centres in the last 10 years and treated with pamidronate and/or TNF-α blockade. 17 patients were treated with pamidronate and/or TNF-α blockade. Out of these 17 patients, 10 were treated with pamidronate alone and showed clinical improvement. Three of the 17 patients were initially treated with TNF-α blockade; two had a positive response, one patient stopped therapy due to minor side effects. Two or one patients were initially treated with pamidonate or TNF-α blockade, respectively, but did not show clinical improvement. Interestingly, switching from pamidronate to TNF-α blockade or vice versa was associated with clinical improvement in these patients. One patient was treated with a combination therapy using pamidronate and TNF-α blockade and showed a good clinical response.

Pamidronate and TNF-α blockade showed a good response in therapy refractory CNO patients. Severe adverse effects were not observed. However, some patients seem to benefit from one of the two therapeutic options in particular. Future studies are needed aiming to identify clinical and/or radiological parameters that might guide the decision to the appropriate treatment in an individual patient.

